# Supplementary figures and images for: Phylogenomic signatures of repeat-induced point mutations across the fungal kingdom
Source: PLoS Biol. 2025 Oct 15;23(10):e3003433. doi: 10.1371/journal.pbio.3003433 (PMC12543289; doi:10.1371/journal.pbio.3003433)

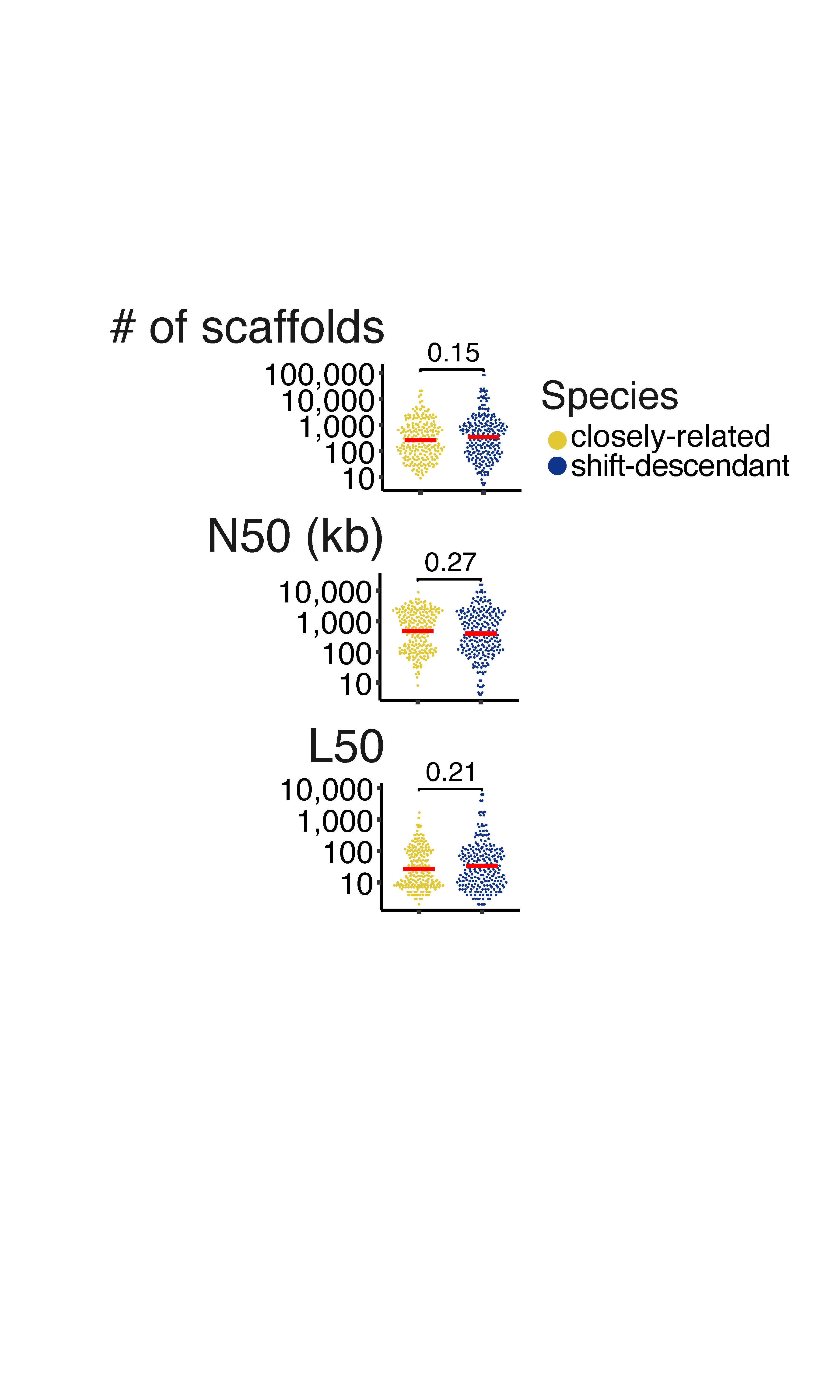

Supplement: S5 Fig — Genome assembly quality given the number of scaffolds, N50 and L50 values for species associated with at a shift in one metric of genome architecture (shift-descendent) compared to close relatives with no shift (closely-related). The data underlying this figure can be found in https://zenodo.org/records/15425698. (TIFF) [file pbio.3003433.s005.tiff]

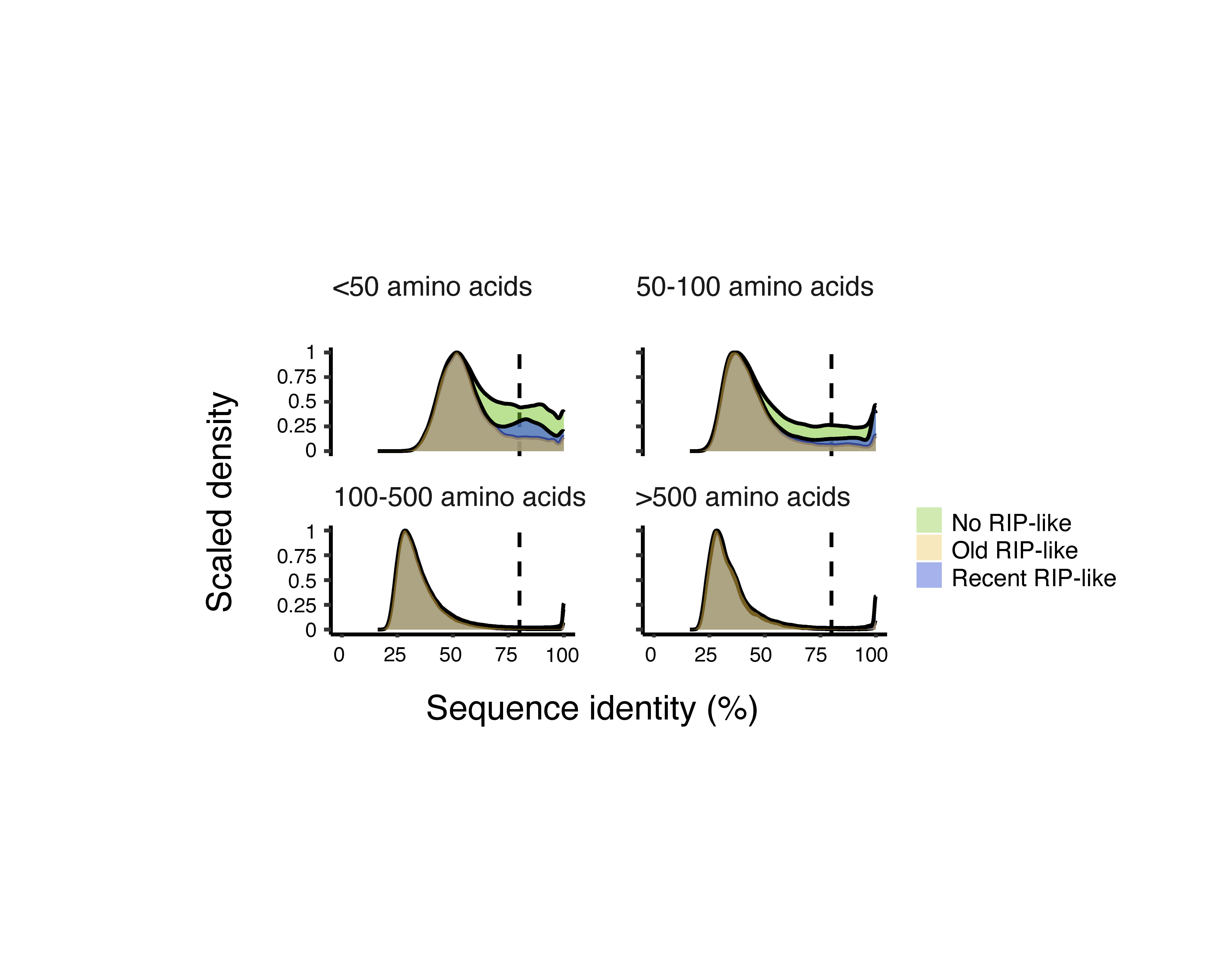

Supplement: S21 Fig — The x-axis shows protein percentage sequence identity calculated from reciprocal blasts. The y-axis is the scaled density given the number of blast hits. Blast hits with sequence length < 50, between 50 and 100, between 100 and 500 or larger than 500 amino acids are represented in the four facets. The data underlying this figure can be found in https://zenodo.org/records/15425698. (TIFF) [file pbio.3003433.s021.tiff]
