# Supplementary material for: Phylogenomic signatures of repeat-induced point mutations across the fungal kingdom
Source: PLoS Biol. 2025 Oct 15;23(10):e3003433. doi: 10.1371/journal.pbio.3003433 (PMC12543289; doi:10.1371/journal.pbio.3003433)
Supplement: Attachment — Submitted filename: Response_to_reviewers.pdf [file pbio.3003433.s047.pdf]

Response to comments to the submitted manuscript [PBIOLOGY-D-25-00319R1]

Editor: *You'll see that reviewer #1 is impressed by the scale of the study, but raises several serious concerns. One is your reliance on assembly size as a proxy for genome size (s/he suggests that you filter for quality or use an orthogonal measure). S/he also says that you need to consider polyploidy/WGD, and questions the clarity and logic behind your K-mer analysis. Reviewer #2 is similarly impressed, but finds the support for the association between genome size and RIP to be rather anecdotal, and the treatment of "lifestyle" unclear. Reviewer #3 is very positive and only has a few minor requests. Reviewer #4 is broadly positive, but s/he wants you to improve the accessibility of the paper, and shares #1's concerns about the K-mer analysis and #2's issue with lifestyle.*

Response: We are grateful for the time and care the reviewers and editor have taken to evaluate our work. We have carefully addressed all major and minor points raised, including those related to genome size estimation, lifestyle classification, and clarification of the *k-mer* analysis. We believe that these revisions have substantially improved the manuscript and strengthened the clarity and robustness of our conclusions. Below, we provide a detailed point-by-point response to the reviewers' comments.

Reviewer #1:

Overview: Badet and Croll analyzed large numbers of fungal genomes to evaluate how transposable elements contribute to genome size. To understand whether the defense of TEs through repeat-induced point mutation (RIP) pathway influence TE abundance and genome size, they catalogued the presence/absence of members in the pathway, finding that the loss of some components are associated with increase in genome size. Further, they developed a kmer based computational method to estimate the extent to which TEs have been deactivated by RIP across the genomes.

Comments: A comprehensive study of repeat composition in fungi is highly welcomed considering how the kingdom has been understudied compared to animals and plants. While examining 1200+ genomes is truly impressive and ambitious and there are some methodological advances, I have several major concerns regarding the methodology and rationale. The most serious has to do with the genome size and repeat content estimates, and second the kmer inference on the mutational signature of RIP. Beyond these there are other concerns with the writing and data presentation that need clarification. Specifically, I found the figure legends to be sparse, often missing key descriptions; additionally several figures do not show what the authors claim they show.

Response: We are grateful for the interest and the pertinent revision requests. We believe that we have implemented now new quality control steps, new analyses and text revisions that satisfy the requests. Details follow below.

Genome size and repeat content estimates: The author estimated genome sizes across 1239 assembled genomes. Genome sizes were estimated based on the assembly size which is well established as a poor proxy of genome size. One of the main reasons which is highly relevant to the study is that repetitive sequences are almost always under-represented due to repeat content being collapsed due to sequence homology. This is especially prone to occurs in deep heterochromatin which has no unique sequences but can contribute to sizeable fractions of the genome (tens to hundreds of Mbs). Genome size inflation can also happen when there are assembly errors that result in multiple haplotypes existing in the same genome (aka

uncollapsed heterozygosity). Different methodologies, assembly softwares and settings will produce different extent of these sources of errors. Further, the fact that the quality and completeness of the assembly appears all over the place (as according to % BUSCO), much of the genome size differences especially within clades likely reflect assembly quality. While the large number of genomes appear at face value to be a real strength of the paper, many of them are likely too poor in assembly quality to be used for this study. The authors should at the very least filter out genomes with obviously poor assemblies (low contiguity, low BUSCO, etc), or better yet find orthogonal ways of measuring genome sizes in a small subset of species to validate the differences, especially the extreme ones between closely related species.

Response: We agree that assembly size is an imperfect proxy for actual genome size, particularly for large and repetitive genomes, which are often underestimated. We also acknowledge that a single genome assembly may not accurately represent species-level variation, as genome size can differ substantially due to factors such as accessory chromosomes and structural variation (e.g., pangenomes).

To evaluate the impact of these limitations in our dataset, we focused on species associated with a non-terminal shift in genome size identified by our method. We searched for additional assemblies for 323 species either directly involved in a shift or closely related to those with a shift. We found between 1 and 7 additional assemblies for 141 species, 81 directly associated with a shift and 60 closely related outgroup species. We then assessed variation in assembly size among these species (see the appended Figure 1). Assembly size varied from -51% to +58% relative to the focal genome, although in ~77% of cases, the difference is under 10%, which could correspond to the presence or absence of accessory chromosomes. Notably, the assemblies used in our study tended to be ~6.8% smaller on average than the alternative assemblies (102/141 species), while for 39 species, newer assemblies were ~8.8% larger on average.

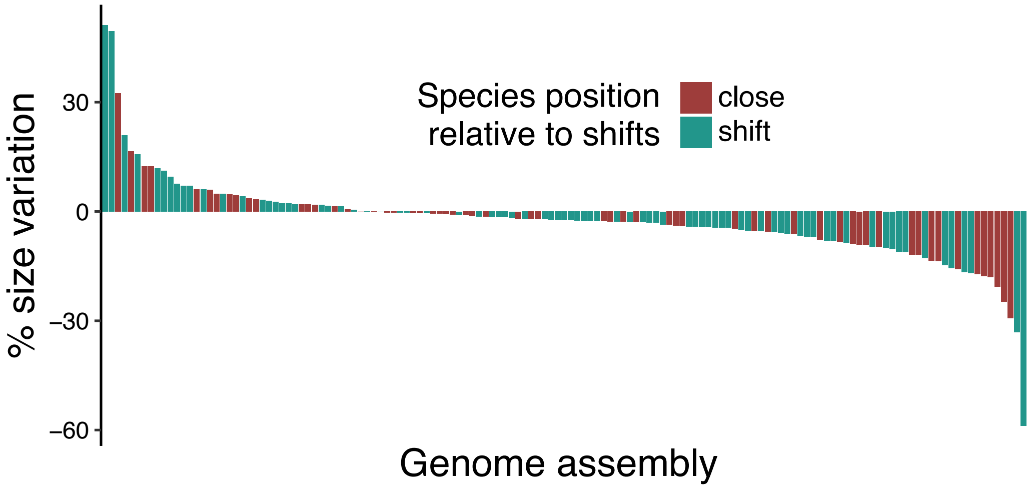

Figure 1: Percentage of variation in size between genome assemblies in our current dataset and additional assemblies available on NCBI

To further evaluate potential confounding effects of assembly quality on inferred genome size shifts, we compared assembly quality metrics (scaffold count, N50, and L50) between species associated with a shift and the closest relatives not associated with one (Figure S5). We found no significant differences. These findings remained consistent when comparing focal and related species within each shift individually (Table S6).

To further control for confounding effects, we also applied the same Brownian motion model used for genome size to three assembly quality metrics: N50, mean scaffold length, and total scaffold count. We detected 23 branches with a significant shift in at least one assembly quality metric. Of these, only 7 co-occurred with a shift in genome size and are now excluded from further consideration. Thus, 54 genome size shifts (88% of the total) remain, which are not associated with changes in assembly quality. We have clarified this point in the Results section and provided additional figures and tables to support these conclusions. We believe this conservative approach to exclude any branch with potential confounding effects between assembly quality and genome size changes significantly strengthens our approach. Altogether, we believe these new analyses strongly support that the identified shifts in genome size are not artifacts of differences in assembly quality.

The authors justify the use of genome assembly estimates by pointing to some degree of phylogenetic signals across at least one of the genome assembly/architecture metrics. First I am not entirely sure what "show phylogenetic signal for at least one of the genome assembly" (line 150) means. Second, it is also not surprising that at least one of the tens of metrics would show some phylogenetic signal, but this criteria strikes me as extremely liberal. Some of this phylogenetic signal on assembly quality may conceivably be driven by technical biases. Labs tend to assemble/sequence closely related species together as part of a paper/project; this could result in more similar assembly qualities as the same methods were used.

Response: As motioned in the previous comment, we now incorporate additional quality checks and focus on shifts in genomic metrics that show no overlap with differences in assembly metrics. The more extensive analyses of alternative genome assemblies, testing for additional assembly quality metrics and excluding any branch with potentially problematic signals should alleviate this significant concern.

The authors report large differences between the major clades; this is likely true (at least qualitatively) since so many genomes were examined as to average out some sources of the noise due to assembly quality. But they also examined fine scale differences in a phylogenetic context leading to their conclusion that genome size changes are closer to tree tips (and therefore recent). High turnover at terminal edges is expected when the values at the tips are highly noisy. By extension, their key result showing that genome size is associated with lifestyle is therefore hard to interpret.

Response: We agree with this concern. We believe the steps outlined in our answers above should significantly alleviate these concerns though. We have now clarified this point and focus only on genome size shifts where we find no co-occurrent shift in genome assembly metrics.

It is well established that whole genome duplication occurred in yeasts (and perhaps several). Whole genome duplication or polyploidy is thought to frequently occur across fungi. The authors should include polyploidy and whole genome duplication as an underlying mechanism driving genome size and gene content differences.

Response: We agree that whole-genome duplication (WGD) and polyploidy are important mechanisms shaping genome evolution, particularly in yeasts, and require consideration. However, there is scant evidence for WGD or polyploidy outside of the Saccharomycotina clade [1–3]. All assemblies included in our study are haploid. Among the 1,239 assemblies analyzed, only *Coprinellus micaceus, Cryomyces minteri, Rhizopus azygosporus*, and *Verticillium longisporum* show at least 25% duplicated BUSCO genes (ranging from 27% to 38.2%). Of these, two (*C. micaceus* and *V. longisporum*) are known diploid species with large genomes.

Importantly, none of the four show a corresponding increase in genome size that would indicate a major shift in genome architecture. These findings suggest that the Ornstein–Uhlenbeck model we employed remains robust even in the presence of such cases, and that WGD or polyploidy is unlikely to be a major confounding factor in our analysis of genome size evolution across fungi. We mention now the issue of polyploidy and WGD as a potential confounding factor and that in our dataset this

<u>Kmer analyses and mutational signature of RIP:</u> The authors designed a pipeline to detect RIP activity in mutating TEs, reasoning that more active RIP should produce more mutated versions of TEs, which can be evaluated looking at the distribution of kmers particularly in repeat rich regions. This seems reasonable, but I had an extremely hard time understanding what is being done here. First the author divides the genome into three sections "coding, non-coding, and repeated sequence". Are these mutually exclusive classes? Figure 5A seems to suggest otherwise and that repeats are either always coding or non-coding. The equations are also poorly explained. f is presumably frequency but what is n? When using f_repeat, are those both the coding and noncoding repeats?

Response: We regret this confusing aspect of the methodology. The three genomic compartments are not mutually exclusive as repeated sequences such as TEs can carry both coding and non-coding sequences. As a result, repeated sequences are indeed either part of the coding or non-coding compartment as shown in Figure 5A. The rationale is that any repeat-directed mutagenic mechanism should yield stronger signatures specifically at repeated sequences. We now clarify the legend of the Figure 5A and the associated text.

It is not clear to me what the expectation should be in the kmer analyses. Kmer distribution will depend on many things including repeat abundance, age of TE amplification, mutation rate, and biases in mutations. Generally, repeated regions should have a highly skewed kmer distribution compared to unique regions as some kmers will have high frequency. If there is elevated mutation rate at TEs via RIP, there should be fewer extremely over-represented kmers, flattening the skew. So if RIP is active kmer frequency at TEs should be closer to kmer frequency at unique regions. The author seems to be suggesting otherwise.

Response: We agree that the expectations from the *k-mer* analysis were not clearly stated. We have revised the relevant section to clarify our rationale. Specifically, we argue that mutagenic mechanisms targeting repeated elements such as RIP should leave strong and predictable signatures in repetitive sequences. To detect these patterns, we designed a genome-wide screen for mutation signatures that does not rely on a predefined RIP motif but instead tests the relative enrichment or depletion of 336 diagnostic *k-mers*. Our approach segments each genome into coding, non-coding, and the non-exclusive category of repeat-associated sequences, allowing us to compare *k-mer* profiles across these compartments. Under active RIP, we expect that specific *k-mers* will be systematically skewed in their frequency in repeated, non-coding regions, relative to other compartments. In contrast, in the absence of directed mutagenesis, the action of neutral drift or random mutation should alter *k-mer* frequencies stochastically, without consistent directional biases. To strengthen our rationale for using *k-mers*, we conducted new analyses to investigate experimental evidence for the effect of RIP mutations on *k-mer* frequencies in *Neurospora crassa* (see next comment and paragraph at page 14 and Figure S18). We now include this rationale in the Results section and clarify the expected signal of RIP versus random mutation processes.

It is also worth pointing out, the age of the TE insertions and normal mutations (not RIP) will also strongly influence the mutational signature that the authors are relying on. Admittedly, I may be

wrong in my interpretation, but in this instance the authors also do not provide a convincing case or benchmarking that their interpretation is correct. The authors need to run some simulations on toy genomes to see how RIP, repeat abundance, age of repeats, and normal mutation rate affect the kmer distributions.

Response: We agree that, at the genome-wide scale, *k-mer* distributions are strongly influenced by factors such as isochore structure and the composition of repetitive elements. We also agree that we had failed to provide a strong connection between actual evidence of RIP and our *k-mer* approach. To remedy this, we have conducted new analyses on *Neurospora crassa* where experimental evidence has shown that Repeat-Induced Point mutation (RIP) introduces thousands of C-to-T and G-to-A mutations within a single sexual cycle, specifically targeting duplicated sequences [4,5]. This mutational process is expected to introduce a distinct bias in *k-mer* frequencies at repeats compared to non-coding or coding regions within the same genome.

To formally assess the impact of RIP on *k-mer* frequencies at repetitive DNA, we now include resequencing data from *N. crassa* engineered strains carrying an artificial duplication introduced prior to crossing [6]. As anticipated, after a single sexual cycle, the duplicated sequences are extensively mutated by RIP, resulting in a marked enrichment of AT-rich *k-mers* specifically within the repeated regions (Figure S18). Crucially, we demonstrate that this shift in *k-mer* composition is tightly linked to RIP activity rather than a general mutational bias, as a parallel cross performed in a *rid1* deletion strain (lacking RIP activity) shows minimal sequence change and no significant alteration in *k-mer* frequencies at the duplicated sequence. We believe that now providing a direct link between *k-mer* frequency and RIP mutations clarifies the biological significance of our *k-mer* approach at inferring RIP activity in individual genomes.

When calculating the non-coding frequency/enrichment, are the repeat compartments classified as noncoding also included? If so, wouldn't much of the elevated kmer frequency signal be driven by the repeats, leading to a deflated repeat enrichment? If the non-coding frequency does not include repeat comparments, wouldn't the coding and non-coding compartments have similar kmer profiles since both are primarily unique sequences?

Response: See comment above. We now clarify that the three genomic compartments are not mutually exclusive, with repeated sequences being either part of the coding or non-coding compartment as shown in Figure 5A. Under a repeat-directed mutagenic mechanism, we expect stronger signatures specifically at repeated sequences compared to coding and non-coding sequences overall. We also note that non-coding regions may include degenerated or unrecognizable ancient repeats, which can contribute to their distinct *k-mer* composition when compared to coding regions, which are further constrained by codon usage. In support of this interpretation, we now provide direct experimental evidence demonstrating the impact of RIP mutations on *k-mer* frequencies. In *Neurospora crassa*, we show that RIP leads to the enrichment of AT-rich *k-mers* specifically at duplicated coding sequences (repeats), but not in an adjacent linker region (non-coding but non-repetitive). This result aligns with our expectations under a repeat-directed mutagenic model.

The author mentions enrichment of AT-rich kmers at repeats as evidence for RIP, but repeating sequences are typically AT-rich anyway.

Response: See comments above. While we agree that repeats are often AT-rich, our *k-mer* approach goes beyond detecting general base composition by identifying specific, reproducible biases in *k-mer* frequencies that are consistent with the mutational signature of RIP. We now

provide experimental data on *N. crassa* to demonstrate that these biases are not simply due to isochore structure or repeat content but are tightly linked to RIP activity. The experimental data shows that AT-rich *k-mer* enrichment occurs specifically in repeated regions undergoing RIP, and not in controls lacking RIP. We believe that our approach provides a functional and mechanistic readout of genome defense activity rather than a descriptive assessment of sequence composition.

<u>Others:</u> *Rapid evolution of TE-silencing genes is well known and the repeated protein adaptation can obscure sequence homology. The consequences of this is multi-fold. First as annotation softwares often rely on sequence homology from other species, rapidly evolving genes may be absent in the annotation. Second, even if they are annotated, the orthology relationship maybe too distant to be called. Can the author rule out the possibility that the missing RIP and TE-silencing genes are either just missing in the annotations or that they are too diverged to be identified based on sequence homology?*

Response: We agree that at this phylogenetic scale, we cannot fully rule out the possibility of misannotation in some genomes, *i.e.* leading to false negatives. Protein homology was inferred using diamond and the *orthofinder* pipeline that accounts for species divergence [7]. To avoid relying on global sequence homology, we now perform a domain search using Pfam HMMs across all proteomes [8,9]. We focus on the DNA methyltransferase domain that is common to both Rid1 and Dim2 (PF000145). We identify a total of 874 assemblies with at least one protein with a PF000145 domain, while 794 species carry either Rid1 or Dim2 as per the initial protein orthology assignment. To investigate if we might be overlooking some species in regard to the presence of those two DNA methyltransferases important for RIP, we reconstruct the phylogenetic relationship of all the proteins with a PF000145 domain (Figure S9). The results reveal clear clustering of the domains according to the DNMT family, with well-defined Rid1 and Dim2 clades. We believe these additional results confirm the observation that RIP-related genes show substantial presence/absence variation among fungi.

As a complementary measure, we also perform genome-wide scans using *tblastn* searching for the two DNA methyltransferases known to be involved in RIP (*Rid1* and *Dim2*). This approach should reveal putative coding sequences that were not annotated in the original assembly and therefore ignored in the current dataset. A total of 18 genome assemblies with no annotated Rid1 or Dim2 homolog had a blast hit with cumulative coverage >60% and >30% average sequence identity to the *Neurospora crassa* Rid1 and Dim2 proteins (see the appended Figure 2). Noticeably, we find that the *Zymoseptoria tritici* assembly, known to lack a functional Dim2 copy, has a region that shares ~34% sequence identity over ~60% of Dim2 protein sequence, illustrating the challenges at inferring protein presence and functionality based on genomic evidence only [10]. Our new analyses suggest that the potential for misannotation is minor compared to the broader presence/absence patterns initially detected.

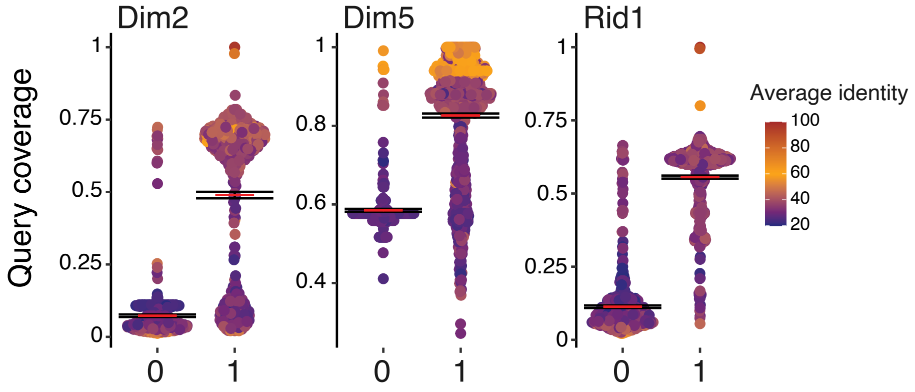

Figure 2: Result of the *tblastn* genome-wide scans using *Neurospora crassa* Rid1, Dim2 and Dim5 proteins. Each dot represents the cumulative coverage (y-axis) and the average sequence identity (color) for translated hits to the query. Hits are reported for assemblies with no (0) or an annotated ortholog to the query (1).

The last section titled "RIP-like mutations associated with DNA-binding proteins" seems highly speculative. The fact that lifestyle has so many more associated orthogroups is suggestive that their genome size and architecture datasets are likely too noisy. In line 340, they also state that the associations are with the mutation signatures, but it does not appear that any of the associations were with the RIP signatures/kmer enrichment metrics from the previous section.

Response: We agree with the need for greater clarity and caution in interpreting these associations. We have now revised the final section to better distinguish correlative findings from mechanistic conclusions. We clarify that while our association analyses broadly covered genome features including size, segmentation, lifestyle, and *k-mer* enrichment, only one orthogroup (OG0000460) showed a strong and direct association with the specific RIP-like *k-mer* enrichment metric described earlier. Note that we now also provide experimental evidence for the impact of RIP mutations of *k-mer* frequencies at repeated sequences (see previous comments). Altogether, we believe the identified DNA-binding protein might play a role in RIP, and we now frame this as a hypothesis supported by correlation, not causality. We also would like to mention that the direct impact of the OG0000460 orthogroup is currently being investigated in two fungi, *Neurospora crassa* and *Podospora anserina*. The text was adjusted to better reflect where uncertainty remains.

The authors argue the Dim2 locus is poorly conserved as support for repeated gene loss, but it could also be that Dim2 translocated multiple times. A cursory google search of Dim2 which the authors claim is nearly absent in Sacchromyctes and Eurotiomycetes show that it is important for RNA processing and ribosome assembly indicating it has a function beyond RIP.

Response: Yes, we agree. We clarified this by adding: “In contrast, the genomic locus surrounding *Dim2* shows poor conservation across the fungal phylogeny. This lack of synteny may reflect recurrent gene loss but could also result from translocations or local rearrangements. The disrupted genomic context in multiple lineages suggest that *Dim2* may be under relaxed selective constraint in many fungi. This is consistent with the broader pattern of loss or pseudogenization observed across ascomycetes and supports the idea that Dim2, while involved in multiple cellular functions, may not be essential in all fungal lineages.”

Not clear what lifestyle changes mean and what increase and decrease means in this context.

Based on methods, this is referring to trophic level.

Response: Added a more detailed description of what we mean by lifestyle directly in the Results section. "We also analyze lifestyles, defined as the functional trophic profile inferred from the overall composition and abundance of carbohydrate-active enzymes (CAZymes) encoded in the genome, which reflects the organism's capacity to degrade different substrates and thus serves as a proxy for ecological lifestyle." In this context, increases or decreases in "trophism values" refer to shifts along principal components summarizing CAZyme repertoires, not direct changes in ecological behavior or habitat.

The title does not accurately describe this paper. First, RIP is the only TE defense relevant to the paper and there are other mechanisms including small RNAs. Second, a "systematic screen" reads like a genetic screen or molecular screen, but instead the "screen" is a genomics pipeline intended to identify RIP activity. Not to say one is better than the other, but the current title is easily misinterpreted.

Response: We changed the title to reflect more accurately the extent of the study: "Phylogenomic signatures of repeat-induced point mutations across the fungal kingdom".

Below are some of the more glaring issues I had with the figures. As mentioned earlier, most of the legends do not adequately describe the figures.

Figure 1E, the branch colours indicating genome sizes are very hard to see. The orange circles are internal nodes seem to have no purpose and obscures the colors of the branches.

Response: Fixed accordingly

Figure 2E is more clear but better color scheme is stil recommended.

Response: Fixed accordingly

Figure 3A is missing color legend for the presence/absence heatmap.

Response: Fixed accordingly

Figure 3B is very confusing and poorly described in the legend. There is a lot of homology between genes (mustard) in Cadophora and tranposons (pink) in blumeria. What are the light purple and green in the RID1 locus? There are faded lines and then also dark blue and red lines. The purpose of this figure is to show synteny. I am clearly missing something, as the two genomic regions do not show the same gene order nor have the same orthologs.

Response: The figure illustrates the fact that *Rid1* (in green) was recently lost in the Blumeria species, as shown per relative synteny at the *Rid1* locus between the two species (in light blue), despite ~100 MY of divergence. We clarified in the legend what the different colours represent.

Figure 4A is poorly explained. What are the pangenome categories? This figure also does not support the statements in which it is invoked such as sentences in lines 217-219.

Response: We clarified the legend: "Proportion of each of the 1,239 proteomes assigned to pangenome categories. Bars represent, for each species, the proportion of its proteome assigned to pangenome categories. Categories include orthogroups present in 80–90% of the

species (soft-core), 50–80% (shell), <50% (variable), and orthogroups found in only one species (unique). The core set of proteins (orthogroups present in >90% of species) is not shown."

Reviewer #2:

Badet and Croll tackle the question of phylogenetic mosaicism in TE defense mechanisms and the associated impact on extant genome architecture. This is a challenging but important question to address, as it is key to fully understand the contribution of TEs to genome architecture under the lens of host-TE arms race. They focus on RIP in the fungal kingdom, and show examples of presence-absence polymorphism in known RIP genes that correlate with genome size variation. Using k-mer enrichment approaches, they map mutational signatures consistent with RIP on the fungal phylogeny and intersect this data with orthogroup presence-absence to infer new determinants of RIP. While the question is important, the methods cutting-edge and the results presented here are interesting, my impression is that some of the results lack the solidity to fully back the article's claims as they are presented now, as detailed below.

My major concern is on the first part of the manuscript, where the authors test their main hypothesis of an association between known RIP components and genome expansion. The authors highlight a 30 Mb expansion signal in one subclade of Leotiomycetes species. Focusing on genes with known function in the RIP pathway, they try to correlate gene presence-absence with the genome expansion outcome. On lines 194-196, it sounds like the loss of Dnmt5 and Dim2 is conserved across the subclade in question, while in fact it is restricted to only some species. While these species are visibly associated with genome size increases (the group of species comprising Blumeris, Golovinomyces, Erysiphe and Oidium), the random walk algorithm identified no shift basal to these species. While the synteny comparison of the RID1 locus in Blumeria graminis compared to Cadophora makes it convincing that it is a real deletion, no single gene absence seems to correlate with genome expansion, and the pattern seems at best correlated to a complex combination of multiple genes. These aspects suggest to me that the sensitivity of the method used to detect shifts might be insufficient to capture substantial shifts in genome size within the subclade of 29 species. On the other hand, while the presence-absence polymorphism of genes is shown for the 29 species and 2 outgroups, the distribution of these genes outside of this focal group of species is not clearly intersected with genome size shifts. Without wider evidence, it is hard to convincingly conclude that there is a causal relationship between their presence and constraints on genome expansion. Overall, the evidence presented seems too anecdotal to sustain the claim that "the loss of RIP components is associated with an 80% (~30 Mb) increase in genome size in ascomycetes (class of Leotiomycetes)".

Response: We agree with the reviewer that the phrasing in the original submission did not sufficiently reflect the complexity of the relationship between RIP component loss and genome expansion, and we have revised the text accordingly.

Specifically, we now clarify that the loss of Dim2 and Dnmt5 is not conserved across the entire 29-species subclade but rather is observed in a subset of species comprising *Blumeria*, *Golovinomyces*, *Erysiphe*, and *Oidium*. These species are indeed associated with a pronounced increase in genome size, but we agree that no single RIP-associated gene loss can alone explain the observed patterns. We have therefore reworded the relevant sections to avoid creating any impression of direct causation and instead emphasize a statistical association between the loss of multiple RIP components and the potential relaxation of TE control mechanisms. Importantly, we have clarified that while the random walk algorithm does not identify a significant shift basal to this clade, it does infer their common ancestor to already possess an enlarged genome, followed by multiple independent contractions and expansions (Fig. 3A). This suggests a punctuated expansion early in the divergence of this group.

To support our interpretation, we also now note that among the 29 Leotiomycete species, only *Cadophora* species, *Lachnellula occidentalis*, *Phialocephala subalpina* and *Rhynchosporium agropyri* retain clear evidence of recent RIP-like activity based on *k-mer* signatures, further supporting the idea that TE control via RIP may have been lost in much of the clade. We agree this observation is not in itself proof of causality, but when considered alongside evidence of high levels of horizontal TE transfer (HTT) events in Blumeria, Erysiphe, and Oidium according to a recent study (1,354 to 4,022 events, [11]), we believe it is reasonable to propose that a breakdown in TE defense mechanisms facilitated genome expansion in this lineage.

We now better articulate the limitations of the data and interpret our findings with greater caution, describing the loss of RIP genes as a likely contributing factor, rather than a sole driver of genome expansion.

The main conclusion of this first section appears to be that the only significant association of genome size shifts along the fungal phylogeny is with changes in lifestyle. Lifestyle is never clearly defined in the results section. Upon reading the methods, my understanding is that it essentially comes down to coordinates along the PCA produced by the CATAStrophy algorithm, based on the presence/absence polymorphisms of carbohydrate-degrading enzymes. Whether other criteria were included in the lifestyle group of variables, like a basic distinction between phytopathogen and non-phytopathogen species, is unclear. If not, I suggest simply referring to trophism instead of lifestyle.

Response: We thank the reviewer for the suggestion. Information about lifestyle in fungi remains scarce and difficult to estimate from genomic data only. We now reformulate "lifestyle" as "trophism" and add more detail about how we estimated it in the Results section: "We also analyze "trophism", defined as the functional trophic profile inferred from the overall composition and abundance of carbohydrate-active enzymes (CAZymes) encoded in the genome, which reflects the organism's capacity to degrade different substrates and thus serves as a proxy for ecological trophism (Supplementary Table S5). We excluded 11 edges for which we also identified a shift in an assembly metric, leaving 177 shifts in genome architecture across 139 edges of the phylogeny (Fig 2AB). Furthermore, we also compared assembly quality between species associated with a shift in genome size to a similar set of closely related species in the phylogeny. We find no differences in either N50, L50 or the scaffold number between the two sets, making variation in assembly quality a very unlikely factor to explain the observed genome size shifts (Supplementary Fig S5)."

In the reconstruction of the global phylogeny, BUSCO genes were selected at random after applying a threshold of minimal occupancy. How does this method account for the differential rates of evolution between species? Most shifts in genome architecture inferred occur near or at terminal branches of the phylogenies, which is arguably the set of branches that is also most likely affected by local changes in average rate of protein-sequence evolution.

Response: The method applied here for tree reconstruction indeed accounts for differences in protein evolutionary rates. First, the concatenated alignment of 100 BUSCO genes is partitioned, allowing each gene or group of genes to be modelled independently. PartitionFinder2 identifies the best-fit substitution model for each partition, capturing protein-specific evolutionary dynamics. These models are then applied during tree inference using IQ-Tree2, which accommodates rate heterogeneity across partitions and sites. This approach ensures that differences in evolutionary rates among proteins do not bias the phylogenetic reconstruction and results in a more accurate species tree. However, we acknowledge that shifts mapping primarily to terminal branches may reflect a combination of sampling and

assembly bias. For this reason, we focused our interpretation on non-terminal edges where such artifacts are less likely to dominate.

Fig 2D: After reading the manuscript multiple times, I still cannot make sense of what this panel is trying to show, and the single sentence in caption was unfortunately not of much help. The x-axis is not clearly labelled, and how shift intensity was aggregated within (what I understand as) clades is unclear. In addition, how this relates to co-occurrence of shifts on single branches is obscure.

Response: We agree that the figure, without further information, is rather confusing. We have now provided a different visual with an improved legend to highlight the fact that all co-occurring shifts are in the Ascomycota (but not Saccharomycotina), although we find no pattern in the directionality of the shifts.

Fig 3A: It looks like the genome size shift is located at the root of the subclade, while from the text it appears restricted to the top 29 tips. Is the genome size shift at the same internal node as the GC shift? I suggest to make arrows point clearly to a horizontal segment of the tree to remove any ambiguity.

Response: The Brownian motion model predict shifts at edges of the tree, so that the 30-fold shift in genome size is indeed restricted to the 29 offspring tips. The shift in GC content was mapped in a subsequent edge of this clade counting 29 tips. We now clarify this in the text "Analyzing five metrics of genome architecture, we identified a total of 229 shifts across 150 edges of the phylogeny".

Fig 3A right: no color code is provided for the heatmap. I suggest using the same one as adopted for the following figure.

Response: Fixed accordingly

Fig 3B: The color code is incomplete. What do light blue and solid green represent should be clearly defined.

Response: We added more information to the legend.

Fig 5C: the color code for AT contents of tetramers seems inverted.

Response: Thank you for spotting it, we fixed it accordingly.

Fig 6A: I understand that the use of an UpSet plot aims to make the point that no orthogroup was significantly associated with more than one feature. However, the fact that no actual intersection is shown on the plot left me wondering why I was looking at redundant information on both axes for a while. The choice of this plot might bring more confusion than clarity, in my opinion.

Response: Thank you for the suggestion, we now provide simple barplots.

L40: a word seems to be missing from this sentence

Response: Fixed accordingly

L534: Genome architecture metrics were log10-transformed, but null values were kept as zeroes. On a log10 scale, that means that null values are equivalent to non-transformed values of 1?

Response: Exactly, note however that there are no values of 1 in the associated genome metrics.

Reviewer #3:

Review of Badet and Croll for Plos Biology 2025

The manuscript presents the association between fungal transposons, transposon-control mechanisms, and genome size, and interprets results in terms of lifestyle. The manuscript is well-written, presents masterfully executed computational biology, and will make a strong impact on fungal comparative genomics and systems biology.

There are few minor issues with the manuscript. One, is the assumption of teleology at several points, which I would recommend be reworded to fit within a more formal Darwinian paradigm. For instance, in the introduction: genome intentionality is suggested by "Genomes evolved...with the primary aim of controlling the expression". Or in the abstract "Genome defenses have evolved to counteract...".

Response: Thank you for the suggestion, we reworded the sentences to avoid suggesting genome intentionality.

The third to last sentence in the abstract is missing a verb.

Response: Thank you for noticing, we fixed this accordingly.

What is the reason for excluding 103 genomes from the initial sample of 1,342?
It appears that provided annotations were used. Should note how annotations were selected and how variable annotation quality could impact some interpretations of gene loss where relevant, and note where existing proteomes were used and where de novo gene detection was done or confirmed for target genes where applicable. Gene and protein are often used interchangeably, which could be made more precise.

Response: We indeed excluded 103 genomes that were redundant (multiple genomes for the same species) or that had missing or partial annotations or that did not fit the 50% BUSCO genes occupancy criteria for phylogenetic inference. We now clarify that in the methods section.

Reviewer #4:

In the manuscript entitled 'A systematic screen for genetic factors underpinning transposon defense systems across the fungal kingdom", T. Badet and D. Croll provide a unique overview of the effects of a defense mechanism called RIP, for Repeat Point Mutation on fungal genome evolution. RIP is of great importance for the evolution of fungal genomes because it is the guardian against the spread of transposable elements, with the consequence that unprotected genomes are much larger than protected ones. In addition, RIP introduces characteristic C to T mutations, creating AT-rich genomic compartments within fungal genomes. Thus, RIP one of the major forces that drives the evolution of fungal genomes. Taking advantage of the massive fungal genome sequencing effort undertaken by the community over the last 15 years, the authors searched 1,211 genomes for RIP traces and genes associated with RIP processing, a first of its kind. Rather than using existing tools to measure RIP-induced mutation bias, the authors have chosen to develop an original and sound Kmers-based method. Rather than considering 'RIP indexes' over sliding windows as in previous published methods, the authors have chosen to compare enrichment in mutation signatures in non-coding and repetitive sequences. This approach is extremely interesting because it allows measurement of RIP dynamics (i.e. RIP signature in non-coding sequences = older RIP and RIP signature in repetitive sequences = newer RIP). This is the most original aspect of the manuscript. The authors also looked for sets of orthologs that would be specifically present in RIP-proficient fungal species. They identified a previously uncharacterized putative zinc finger transcription factor.

Overall, this study is of general interest and suitable for publication in PLOS Biology. The results are sound and support the conclusions. However, in my opinion, a number of important points need to be addressed before this manuscript can be considered for publication.

Major points:

- I find this article very "technical", considering the broad intended readership (pending publication in PLOS Biology). The text should be more accessible to non-bioinformaticians. This would require a better description of methodology used at each stage of the analysis. It would be good to explain the main operating principles of the tools used (e.g. BUSCO, what kind of information does it give? And how is it obtained?).

Response: We now provide a broader description of the methodology where relevant in the Results section.

- The figures in the article appear attractive at first glance. However, they are difficult to read and quite small (although it is possible to zoom in and still read the output). It would be useful 1) to enlarge the visuals (no more than 2 graphs per line) and 2) to detail the associated legends (defining precisely the information shown - e.g. "Repetitive vs Non repetitive" Fig. 1D). Note an error in the Fig 1 legend (part C).

Response: We thank the reviewer for the careful examination of the manuscript. We reshaped figures accordingly.

- The link between "genome architecture" and "lifestyle" is difficult to grasp. The paragraph entitled "Shifts in genome size associated with lifestyle changes" should be rewritten for those not familiar with the Ornstein-Uhlenbeck process. In particular, the metrics of genome architecture and the criteria chosen to define this lifestyle should be made explicit.

Response: We made more explicit what we mean by lifestyle in the Results section and reworded it as "trophism" to more accurately reflect what we are looking at.

- Benchmarking of K-mer RIP measurement method: The method developed in this study to measure the mutations installed by the RIP is interesting. However, the result is sometimes a little puzzling compared to what one would expect for species where the RIP is known to be active. It would therefore be useful to compare the results of RIP efficiency analysis via K-mer with those of existing methods such as The RIPper (DOI: 10.7717/peerj.7447). This should be done on a small number of species, e.g. those where the existence of the RIP has been shown experimentally, to test whether the K-mer method can detect recent RIP in species where this occurs readily at each cross.

Response: We thank the reviewer for the suggestions, and as mentioned above we now include results on resequencing data from engineered strains of *N. crassa* carrying an artificial duplication introduced prior to crossing [6]. The results are summarized in Figure S18 and highlight similar shifts in *k-mer* composition after a single round of RIP activity as those observed in our genome-wide screen across the fungal kingdom. We believe that we now provide a more direct link between *k-mer* frequency and RIP mutations justifying the validity of the *k-mer* approach at inferring RIP activity in individual genomes.

- K-mer enrichment in repetitive sequences. The large number of additional tables does not make K-mer enrichments easy to read, nor does it provide a clear overview. In the text (lines 262-264), the authors draw attention to a set of ten k-mers enriched >2-fold in non-coding sequences, that would be a good proxy to detect RIP signatures. It would be interesting to find these K-mers in a short, readable, non-supplementary table. In the same line, why did you choose this set of K-mers in Fig. 5B? Do any of them correspond to the 10 k-mers mentioned above? In the legend to Fig. 5E and in the text (lines 293-294), it is stated that a value greater than 0.5 was consistent with recent RIP activity. This result deserves to be better explained.

Response: We now provide a clearer summary of *k-mer* enrichments to address readability concerns. While Table S12 includes the raw enrichment values across all *k-mers*, genomes, and sequence categories (noncoding and repetitive), we acknowledge that the large volume of data may obscure key patterns. We added two columns in Table S12 that summarises the proportion of species in the dataset with the focal *k-mer* being >2-fold enriched at either non-coding or repeated sequences. To improve accessibility, we also added a new column in Table S13 that summarizes the estimated RIP status of each genome. This is based on the number of *k-mers* showing >2-fold enrichment in both noncoding and repetitive sequences. This pattern is consistent with RIP-like mutational signatures and the rationale behind has been clarified in the Results section. To improve interpretability of Figure 5B, we now restrict it to the *k-mers* with the 20 highest enrichment values in repeats. This subset includes several of the ten *k-mers* previously highlighted. These refinements aim to provide both a comprehensive dataset for exploration (Table S12) and more intuitive summaries of RIP-related trends (Table S13 and Figure 5B).

Minor points:

Abstract:
Lines 40-41: this sentence does not make sense. Please rephrase.

Response: Rephrased as "Then, we performed a phylogeny-aware association study to identify gene functions associated with RIP-like mutation signatures".

Introduction:
Lines 80-81: This sentence seems to imply that a package consisting of DNA methylation and a complete histone post-translational modification system (i.e. equivalent to that of animals) is either completely conserved or completely lost. This is not the case. This sentence needs to be edited accordingly.

Response: Rephrased as "While many fungi share conserved elements of the machinery for DNA methylation, histone modifications, and RNAi-associated pathways, these machineries show also considerable variation in their components across fungal lineages".

Lines 93-94: This sentence is unclear. Explain "that segregate presence absence in the phylum".

Rephrased and developed this section to include the complex relationship between RIP and presence/absence of the different methyltransferases: "In *N. crassa*, two mechanistically distinct RIP pathways have been described. One requires the Rid1-encoded cytosine methyltransferase and targets repetitive sequences themselves, while a second pathway dependent on the cytosine methyltransferase Dim2 and the histone H3 lysine 9 methyltransferase Dim5, targets the single-copy regions flanked by the repeats. These two pathways likely operate with different dependencies, genomic targets, and potentially distinct biological roles, yet this dual origin and its implications are poorly understood. Noticeably, the genes encoding Dim5, Rid1 and Dim2 have been lost multiple times in the phylum".

Results:
Line 109: Why did you choose some Stramenopiles as outgroup species, since they are so far away from the eumycetes?

Response: We argue that despite their phylogenetic distance, stramenopiles and eumycetes share similar genomic features such as compartmentalization and recent TE expansions. We add more context to the Results section: "Although distantly related, these lineages were chosen as outgroups to help distinguish fungal-specific patterns from those shared more broadly across eukaryotes."

Line 214: This statement is somewhat exaggerated: the cause or effect of RIP is still very poorly understood. The wording should be revised.

Response: We toned down the sentence as "In *N. crassa*, RIP is associated with both DNA and histone methylation, though the precise mechanisms remain unclear".

Lines 228-229: This is also a bit of an overstatement: is some fungi, RIP is associated to DNA methylation, but for others, there is no detectable DNA methylation but RIP is efficient. Also, RIP in N. crassa is independent of recombination pathway. n addition, two RIP pathways have been described in N. crassa, the RID-dependent one that targets repeats and the DIM-2-dependent one that targets the linker between repeats. Why was this dual origin never mentioned or discussed in this manuscript?

Response: We are grateful for the commentary and now incorporate more information about this duality. In "Repeat-targeted mutations have been associated with epigenetic modifications and DNA repair machinery, although the underlying mechanisms vary across fungi. For

example, while DNA methylation is involved in some species, others show efficient RIP activity in its absence, and RIP in *N. crassa* operates independently of the canonical recombination machinery" and "In contrast, the genomic locus surrounding Dim2 shows poor conservation across the fungal kingdom. This lack of synteny may reflect recurrent gene loss but could also result from translocations or local rearrangements. The disrupted genomic context in multiple lineages suggests that Dim2 may be under relaxed selective constraint in many fungi. This is consistent with the broader pattern of loss or pseudogenization observed across Ascomycetes and supports the idea that Dim2, while involved in multiple cellular functions, may not be essential in all fungal lineages.". We also provide additional context in the introduction "In *N. crassa*, two mechanistically distinct RIP pathways have been described. One requires the Rid1-encoded cytosine methyltransferase and targets repetitive sequences themselves, while a second pathway dependent on the cytosine methyltransferase Dim2 and the histone H3 lysine 9 methyltransferase Dim5, targets the single-copy regions flanked by the repeats. These two pathways likely operate with different dependencies, genomic targets, and potentially distinct biological roles, yet this dual origin and its implications are poorly understood. Noticeably, the genes encoding Dim5, Rid1 and Dim2 have been lost multiple times in the phylum".

Lines 271-273: I'm not sure I understand the meaning of this sentence. It needs to be clarified.

Response: We changed to "However, we also identified 68 *k-mers* that are enriched >2-fold in non-coding sequences compared to coding sequences, and simultaneously enriched >2-fold in repetitive regions compared to non-repetitive regions."

Line 296: According to table S12, 134 species show a ratio strictly greater than 0.5, whereas the text indicates 130 species. Is there an additional selection step? If so, please indicate which one and which species are concerned.

Response: Indeed, 134 species have a ratio greater than 0.5, and four species have less than 10 *k-mers* enriched in both compartments (column n_both in Table S12, *Arthrobotrys flagrans*, *Botrytis cinerea*, *Fusarium langsethiae* and *Penicillium coprophilum*).

Line 314: Are those three methods described in Mat & Meth section?

Response: The three models are described in the method section as: "For each trait, we reported the associated orthogroups and Pfams given the terminal, simultaneous and subsequent association tests performed by *treeWAS* (Supplementary Table S13)."

Lines 399-401: This statement appears to be incorrect. In P. anserina, no functional link has yet been established between i) RIP and recombination (RIP occurs prior to meiosis), ii) undetected DNA methylation and detected RIP mutations. Isn't that the RIP in T. reesei you are referring to?

Response: We are grateful for the correction, "not" in the sentence was indeed a typo error and has now been corrected as "This is highlighted by the fact that in *P. anserina* the mild RIP-like mutations observed upon sexual recombination are independent from cytosine methylation of the targeted repeats, in contrast to *N. crassa*".

Figures and Figure Legends:
Fig 1: Mucoromycota and Chytridiomycota are present in 1A but not in 1B, why is this? 1B: define RC.

Response: We now provide annotations for the different classes present in Figure 1A-C. We now also define the different abbreviations in the figure legend: “Repeats are classified as Rolling-circle (RC), Long interspersed nuclear element (LINE), Short interspersed nuclear element (SINE), Long terminal repeats (LTR), DNA transposons (DNA) or unknown.”

1C: What is the purpose of this figure? What does it show? 1F: x-axis labels should be defined in the legend

Response: We now provide more detail in the legend “(C) Heatmap showing the number of sequences sharing more than 95% sequence identity over 100 bp, 1 kb, 5 kb and 10 kb across the 1,239 genomes. Only a small fraction of genomes carries multiple >5 kb repeats with >95% sequence identity. Genomes with >1,000 hits of >95% sequence identity were also among the top 25% largest genomes (31 out of 37 genomes).” and define labels in Figure 1F in the legend “Assembly metrics include the average scaffold length (avgLen), percentage of complete BUSCO genes (complete), GC isochore percentage, L50 (smallest number of contigs whose length sum makes up half of genome size), length of the largest scaffold in base-pairs (maxLen), median scaffold length in base-pairs (medianLen), length of the smallest scaffold in base-pairs (minLen), number of GC segments genome-wide (segmentation), N50 (sequence length of the shortest contig at 50% of the total assembly length), number of annotated proteins (proteins), number of scaffolds (seqCounts) and genome size in base-pairs.”

Fig. 2. B: What does the set size scale correspond to? C: What is the unit of the Y-axis ?

Response: We reshaped the figure to clarify. Set size is now labelled as “number of shifts” and panel C is now representing $\log_2$ fold-change values.

Fig. 4. A: Where are the "singletons" on this graph? Do they make up the proportion missing to reach 1 on the y-axis?

Response: We now call singletons “unique” in the Results section to match the Figure 1A. We also define the different pangenome categories in the legend: “Proportion of each of the 1,239 proteomes assigned to pangenome categories. Bars represent, for each species, the proportion of its proteome assigned to pangenome categories. Categories include orthogroups present in 80–90% of the species (soft-core), 50–80% (shell), <50% (variable), and orthogroups found in only one species (unique). The core set of proteins (orthogroups present in >90% of species) is not shown.”

C: Color legend? How should we read the violin plot?

Response: We reshaped the figure to show the total number of RIP-related genes as colors and provide the legend. Each dot represents a genome and its genome metric value. The heatmap represents the repertoire of RIP-related genes in the set of genomes in each of the violins.

D. It's not clear why these five protein families were chosen for this heatmap. Why are they RIP-related proteins? It's clear for part B, but not for part D. Proteins involved in recombination or DNA repair are ubiquitous and their presence/absence is not necessarily functionally related to RIP. So, I'm not sure this part of the figure is of interest.

Response: Our rationale for including PFAM domains related to DNA repair, histones, meiosis, heterochromatin, and RNAi in Figure 5D is that these protein families are broadly relevant to genome defense mechanisms and the biology of RIP. While we agree that some components

such as DNA repair proteins are ubiquitous, they are nonetheless mechanistically linked to mutagenic processes like RIP, which occurs during the sexual cycle. The inclusion of these categories aims to provide a broader view of potential molecular factors associated with the presence or absence of RIP-like signatures across genomes. We have now clarified this rationale in the Results section with “These categories were selected because they represent core components of genome defense systems and can be functionally linked to RIP, which occurs during the sexual cycle and involves interactions between chromatin state, DNA damage response, and transcriptional silencing.”

References

1. Ma LJ, Ibrahim AS, Skory C, Grabherr MG, Burger G, Butler M, et al. Genomic Analysis of the Basal Lineage Fungus Rhizopus oryzae Reveals a Whole-Genome Duplication. PLoS Genet. 2009;5: e1000549. doi:10.1371/JOURNAL.PGEN.1000549
2. Albertin W, Marullo P. Polyploidy in fungi: evolution after whole-genome duplication. Proceedings of the Royal Society B: Biological Sciences. 2012;279: 2497–2509. doi:10.1098/RSPB.2012.0434
3. Wolfe KH, Shields DC. Molecular evidence for an ancient duplication of the entire yeast genome. Nature 1997 387:6634. 1997;387: 708–713. doi:10.1038/42711
4. Selker EU, Stevens JN. DNA methylation at asymmetric sites is associated with numerous transition mutations. Proceedings of the National Academy of Sciences. 1985;82: 8114–8118. doi:10.1073/PNAS.82.23.8114
5. Gladyshev E, Kleckner N. DNA sequence homology induces cytosine-to-thymine mutation by a heterochromatin-related pathway in Neurospora. Nat Genet. 2017;49: 887–894. doi:10.1038/ng.3857
6. He Z, Wu N, Yao R, Tan H, Sun Y, Chen J, et al. RID is required for both repeat-induced point mutation and nucleation of a novel transitional heterochromatic state for euchromatic repeats. Nucleic Acids Res. 2025;53: 263. doi:10.1093/NAR/GKAF263
7. Emms DM, Kelly S. OrthoFinder: Phylogenetic orthology inference for comparative genomics. Genome Biol. 2019;20: 466201. doi:10.1186/s13059-019-1832-y
8. Finn RD, Bateman A, Clements J, Coggill P, Eberhardt RY, Eddy SR, et al. Pfam: the protein families database. Nucleic Acids Res. 2014;42: D222. doi:10.1093/NAR/GKT1223
9. Finn RD, Clements J, Eddy SR. HMMER web server: Interactive sequence similarity searching. Nucleic Acids Res. 2011;39: W29-37. doi:10.1093/nar/gkr367
10. Moller M, Habig M, Lorrain C, Feurtey A, Haueisen J, Fagundes WC, et al. Recent loss of the Dim2 DNA methyltransferase decreases mutation rate in repeats and changes evolutionary trajectory in a fungal pathogen. PLoS Genet. 2021;17: e1009448. doi:10.1371/journal.pgen.1009448
11. Romeijn J, Belaunde IB, Seidl MF. Extensive horizontal transfer of transposable elements shape fungal mobilomes. bioRxiv. 2025; 2025.06.16.659975. doi:10.1101/2025.06.16.659975
