# Supplementary material for: Phylogenomic signatures of repeat-induced point mutations across the fungal kingdom
Source: PLoS Biol. 2025 Oct 15;23(10):e3003433. doi: 10.1371/journal.pbio.3003433 (PMC12543289; doi:10.1371/journal.pbio.3003433)
Supplement: Attachment — Submitted filename: Badet_and_Croll_response2reviews.pdf [file pbio.3003433.s049.pdf]

Response to comments to the submitted manuscript [PBIOLOGY-D-25-02318R2]

Editor:

On reviewer number 1:

1) I agree that there is something odd about the color scheme in Figure 3A. I think that perhaps the units are not the same as in Figure 2E. Figure 2E is in Mb and I think 3A might be proportional to the maximum genome size which explains why the basal branches are "cold" and the max value is 100 and not 1,000. This is minor but does need to be clarified.

Response: We apologize for the confusion. As mentioned above, the shifts in genome size and the branch color scheme in Figure 3A represent two independent measures. The 30-fold increase in genome size was inferred using the OUshifts approach under a Brownian motion model with an Ornstein–Uhlenbeck process, while the branch colors show the maximum likelihood reconstruction of ancestral genome sizes along the tree. To clarify this, we have updated the figure legend to read: "Branch colors represent the maximum likelihood reconstruction of genome size across the phylogenetic tree." Additionally, we adjusted the color scale to ensure consistency with the units shown in both figures.

2) I agree authors should look for the two upstream genes in Fig 3B and perhaps add them to the panel. But i think their overall interpretation does not differ from Reviewer #1: these regions are highly rearranged that potentially indicates relaxed purifying selection.

Response: We now specify in the text the status of the two genes upstream of *Rid1* in the *Blumeria* assembly. Precisely, the gene that directly flanks *Rid1* in *Cadophora* is absent in *Blumeria* while the second has a homolog on another scaffold. This now reads as "Five of the ten genes flanking *Rid1* in *Cadophora* (five upstream and five downstream) were found on the same *B. graminis f. sp. tritici* scaffold. Three of the genes were absent from the assembly, while two were located on other scaffolds. Homology links between translated sequences in the synteny plot between the two scaffolds indicates that, despite local conservation around the Rid1 locus, B. graminis f. sp. tritici clearly lacks the RIP-essential gene (Fig. 3B)."

I looked over comments from reviewer number 4 and I feel they were reasonably addressed. The major comment regarding the Kmer frequencies was addressed nicely with the mutant analysis. Other comments were mostly overlapping with other reviewers to my eye.

Response: We are very grateful for the editor's time and help addressing the comments.

Reviewer #1:

The updated manuscript is much improved. The authors have addressed many of my concerns. The restructured section on the kmer frequency analyses, in particular, is substantially improved on clarity and scientific veracity. The incorporation of kmer analyses on the experimental dataset is intelligent, cogent, and commendable.

Response: We are very grateful for the reviewer's comments, which greatly improved the manuscript, and we are pleased that the revised version has addressed most of the concerns.

Regarding the genome size estimates, I agree with the author's assessment that there are broad phylogenetic signals even if individual estimates are imperfect. I also agree that these signals are sufficient for the paper's analyses. However, as to not set a bad precedent for others to equate assembly size with genome size, I strongly recommend that the authors should include a clear statement in the manuscript indicating that the genome size estimates presented are genome assembly sizes which are imperfect and typically poor approximations (and usually underestimates) of the true genome size.

Response: We now add to the discussion the statement: "However, it should be noted that genome size was inferred from assembly size, which is inherently an imperfect proxy and often underestimates the true genome size."

Regarding the result of 30-fold genome expansion, I may be missing something but this massive increase does not seem to be reflected in the color of the phylogenetic branches in Figures 2E or 3C. It's a bit hard to tell for the former due to obstructing graphical elements, but for the latter, the branch barely changes colour and remains relatively gray even though an increase this large should result in the branch becoming very red.

Response: We apologize for the confusion. Shifts in genome size and the branch color scheme on the tree represent two independent measures. The 30-fold increase in genome size was inferred using the OUshifts approach under a Brownian motion model with an Ornstein–Uhlenbeck process, whereas the branch colors indicate the maximum likelihood reconstruction of ancestral genome sizes along the tree. We now specify in the figure's legend: "Branch colors represent the maximum likelihood reconstruction of genome size across the phylogenetic tree." We also changed the color scale in both figures and increased the offset of the other graphical elements to increase readability.

For figure 3B, the plot is now better labeled, but it is still not clear to me that the correct orthologous region was identified. Many of the RID1 locus genes in Cadophora have no homology to the "syntenic" region in Blumeria. Reciprocally, many of the Blumeria genes also have no homology to the supposed syntenic region in Cadophora. Based on these tracks, it can only be concluded that the RID1 locus in Cadophora is highly

rearranged compared to Blumeria. Importantly, two genes upstream of Rid1 (green) also have no homology, so one interpretation could be that RID1 and the two upstream neighbors translocated into or out of this region. Can the orthologs of the two genes upstream of RID1 be found elsewhere in the genome?

Response: We apologize for the misunderstanding. We now clarify in the legend how synteny was inferred. Precisely, we used *mmseqs* to search for translated hits between *Blumeria* and *Codophora* assemblies (https://github.com/soedinglab/MMseqs2). The scaffold 3 in *Cadophora* where a full copy of *Rid1* is present showed best reciprocal hits to the *Blumeria* scaffold 6 with a total alignment length of 139,935 amino acids for an average identity of 56%. The Figure 3B illustrates these translated hits between the two scaffolds in the 6 possible frames. The synteny clearly shows that hits represent annotated genes in both assemblies and that the *Rid1* ortholog is missing in the *Blumeria* assembly. Regarding the genes flanking *Rid1* in *Cadophora*, we now specify in the text "Five of the ten genes flanking *Rid1* in *Cadophora* (five upstream and five downstream) were found on the same *B. graminis f. sp. tritici* scaffold. Three of the genes were absent from the assembly, while two were located on other scaffolds. Homology links between translated sequences in the synteny plot between the two scaffolds indicates that, despite local conservation around the Rid1 locus, B. graminis f. sp. tritici clearly lacks the RIP-essential gene (Fig. 3B)."

Lines 257: The heading "Genes likely to impact RIP were lost in fungi" implies that all fungi lack these genes, when the authors mean there are recurrent lineage-specific losses of the genes.

Response: We changed the heading to "Recurrent lineage-specific losses of genes likely to impact RIP in fungi".

The authors switch between different taxonomical hierarchies, sometimes using phylum, other times using class or genus. For the mycologists, this might all seem very natural, but this will be quite confusing for a more general audience.

Response: We added along the text the taxonomical level when possible (phylum, subphylum, order, class).

In the abstract (lines 44-46) and discussion (lines 449-452) the authors imply a very close relationship between RIP and genome size. However, based on their results, this relationship seems to be relevant to ascomycota where there is clear RIP signatures, and perhaps leotiomycetes which has a branch with 30-fold increase in the absence of RIP. However, a glaring counter point to this pattern is the Saccharomycetes which has small to modest genome sizes but completely lack the RIP genes. The author acknowledges this later in the discussion (lines 499-502), pointing to trophism as a better predictor. Therefore the tone of the abstract must be reconsidered as to not give

the false impression that RIP or TE regulating mechanisms drive genome size across the kingdom.

Response: We rephrased the abstract as “Our findings reveal the multifaceted drivers of genome defense systems and their close ties to genome size evolution in fungi, particularly in lineages with evidence for recent RIP activity, highlighting how proximate molecular mechanisms can shape genome evolution on deep phylogenetic scales.”

Reviewer #2:

The revision adequately addresses all my comments.

Response: We are grateful for the reviewer’s time and helpful comments.
